# Challenges of Analysing Gene-Environment Interactions in Mouse Models of Schizophrenia

**DOI:** 10.1100/tsw.2011.128

**Published:** 2011-07-07

**Authors:** Peter L. Oliver

**Affiliations:** MRC Functional Genomics Unit, Department of Physiology, Anatomy and Genetics, University of Oxford, Oxford, UK

**Keywords:** mouse, mutant, schizophrenia, prenatal stress, behaviour, HPA axis

## Abstract

The modelling of neuropsychiatric disease using the mouse has provided a wealth of information regarding the relationship between specific genetic lesions and behavioural endophenotypes. However, it is becoming increasingly apparent that synergy between genetic and nongenetic factors is a key feature of these disorders that must also be taken into account. With the inherent limitations of retrospective human studies, experiments in mice have begun to tackle this complex association, combining well-established behavioural paradigms and quantitative neuropathology with a range of environmental insults. The conclusions from this work have been varied, due in part to a lack of standardised methodology, although most have illustrated that phenotypes related to disorders such as schizophrenia are consistently modified. Far fewer studies, however, have attempted to generate a “two-hit” model, whereby the consequences of a pathogenic mutation are analysed in combination with environmental manipulation such as prenatal stress. This significant, yet relatively new, approach is beginning to produce valuable new models of neuropsychiatric disease. Focussing on prenatal and perinatal stress models of schizophrenia, this review discusses the current progress in this field, and highlights important issues regarding the interpretation and comparative analysis of such complex behavioural data.

## INTRODUCTION

It is well established that psychiatric disorders contain a strong genetic component; for example, heritability estimates for schizophrenia typically range from 60 to 80%[1]. The aetiology of such disorders is highly complex, however, and although many putative susceptibility genes have been identified based on genetic epidemiological studies, environmental factors undoubtedly play a major role[2,3,4,5]. Indeed, it has been proposed that heritability rates themselves are highly inaccurate as environmental differences between individuals are not taken into account[6]. Such nongenetic risk factors are known to arise from a wide range of sources (reviewed in Brown[7]). For example, psychiatric disease is frequently associated with specific episodes during pregnancy that may cause stress, including factors as diverse as war, famine, and environmental disasters[3,8]. Prenatal infections such as influenza have also been associated with schizophrenia and neurodevelopmental disorders from retrospective studies, and have since been confirmed by utilising maternal serum archives[9,10]. Postnatal life events are also major risk factors for schizophrenia. For example, increased rates of neuropsychiatric disorders are reported in children brought up in an urban environment, with this "urbanicity" risk factor being replicated in multiple international studies[11,12]. Cannabis use during adolescence is also known to influence the onset of psychotic episodes, although the mechanisms linking recreational drug use and neurodevelopment are still unclear[13,14]. Such environmental factors are often termed "stressors"; the assumption is that maternal exposure to such events activates pathways such as the hypothalamic-pituitary-adrenal (HPA) axis, which ultimately influences early synaptic development via as-yet-unknown mechanisms[8,15,16]. Interpretation of such studies can be difficult, however, as data are often based on the recollection of an event that occurred many years ago. Furthermore, the adverse effects experienced by an individual will be influenced by a number of factors, from their personality to their social-economic situation, in addition to any genetic predisposition[8]. Therefore, to better understand the pathogenesis of psychiatric disease, there is a need to establish well-controlled analyses of gene-environment interactions[17,18]. Although such studies are only in their infancy, the use of mutant mice is already demonstrating that the synergy between genetic lesions and factors influencing early neurodevelopment can be effectively modelled *in vivo*. This review will focus on those experiments that have combined prenatal/perinatal and genetic factors as neurodevelopmental models of schizophrenia.

## METHODOLOGICAL CONSIDERATIONS IN MODELLING GENE-ENVIRONMENT INTERACTIONS

Using animal models of environmental stress provides an opportunity to not only control and vary the stressor itself, but also to monitor the outcome in multiple offspring at a number of developmental time-points on an essentially identical genetic background[16,18]. This provides obvious advantages over retrospective human studies; however, in the complex field of modelling neuropsychiatric disease, with numerous hypotheses to examine and multiple parameters to consider, our current understanding of the mechanisms that underlie environmental stress is still very poor. Considering prenatal/perinatal stress models in rodents, a significant number of studies have been carried out in wild-type mice and rats illustrating how nongenetic factors can cause significant neurodevelopmental and behavioural effects[16,19,20]. Although many general themes emerge, a lack of methodological standardisation has meant that comparison between individual datasets is problematic. Nevertheless, these studies have revealed that when choosing a stress paradigm most relevant to human psychiatric disease, there are many factors that need to be taken into account.

1. **Type of Stressor** — Depending on the aim of the study, the stressor itself should cause measurable stress to the pregnant dam without causing overt adverse effects. For example, methods such as restraint, saline injection, or bright light are commonly used and have been shown to significantly influence the HPA axis based on corticosterone levels[16,19,20]. However, a blunting of the HPA response has been noted when a particular stressor is applied at a predictable time of day[21]. Consequently, randomised protocols are now more frequently applied using a varied range of stress paradigms to maintain the "unpredictable" nature of the method[22,23]. Although such approaches are deceptively simple to apply to rodents, some argue that environmental factors observed in human studies, such as prenatal infection and psychosocial stress, are more valid. Therefore, many experiments use the prenatal immune challenge as an effective prenatal paradigm, typically utilising lipopolysaccharide (LPS) as model of bacterial infection or polydI:dC, a synthetic double-stranded RNA analogue, to mimic viral infection[10,24].

2. **Timing of Stressor** — It is also important to address when and for how long the stressor is applied. This becomes a difficult task when extrapolating rodent studies to humans as key neurodevelopmental milestones generally occur much later in gestation in rodents[15]. Yet, thanks to detailed anatomical studies, it is possible to map such events; for example, prenatal stress applied in the final few days of gestation in mice (E16.5–17.5) equates to the beginning of the second trimester in humans[25]. Although cumulative data do point to early to mid-gestation as the stage of human development most susceptible to prenatal stress[26,27,28], studies in rodents have shown that adverse effects at a range of prenatal stages can generate abnormal behaviour in adulthood[29,30,31]. Early postnatal neurodevelopmental events in rodents are also vital for shaping future outcomes, particularly as such a large proportion of the synaptic connections and neuroanatomical maturation occurs during the first few weeks after birth. Indeed, the first and second weeks after birth in the mouse are still extrapolated to the human second trimester[25]. In addition, it is important to consider postnatal brain development in humans. While structures such as the hippocampus are fully formed by approximately 2 years of age, the amygdala continues to develop for another 20 years, suggesting that stress even after adolescence could play a direct role on neurodevelopment and the subsequent induction of psychiatric disease[8,15].
3. **Sex-Specific Effects** — As sex hormones play an important part in brain development, it is perhaps unsurprising that male and female rodents have been shown to display differing susceptibility to prenatal stress. For example, it has been demonstrated that male mice are more sensitive to the effects of variable prenatal stress on depressive behaviour, learning and memory, and the long-term responsiveness of the HPA axis[29,32]. As a consequence, it is clear that any subsequent behavioural analysis from such studies must be split into same-sex groups. Although the mechanisms that bring about this phenomenon are unclear, it is interesting that, in humans, it has been reported that the male foetus is more susceptible to neurodevelopmental complications than the female[33,34].
4. **Maternal Effects** — In part due to the considerable amount of neurodevelopment that continues in rodents prior to weaning, the influence of maternal behaviour on the offspring must not be underestimated. Importantly, prenatal stress itself has been shown to influence maternal behaviour of the stressed dams. Although current data on this phenomenon are somewhat conflicting, studies have shown that maternal stress can significantly alter nursing activities such as licking and suckling that can both significantly affect future behaviour of the neonates[35,36]. One alternative is to use a randomised cross-fostering method, whereby offspring from stressed dams are fostered by nonstressed mothers; however, examination of this method has shown that there are still discrepancies in the amount of time spent by foster mothers with offspring subjected to prenatal stress vs. control pups[35]. These data demonstrate that maternal behaviour could potentially confound behavioural studies if not considered.

Although it may not be practical to take into account all the factors above in a single study, such experiments in wild-type rodents have begun to reveal the important link between early life "programming" and neurological development. Indeed, in addition to anxiety and memory deficits, prenatal stress has been shown to cause behavioural outcomes relevant to psychiatric disorders such as abnormal sensorimotor gating or prepulse inhibition (PPI) responses[22]. However, a potentially more valuable approach to understanding the mechanisms that underlie complex neurodevelopmental disease would be to consider both genetic and nongenetic factors in a single experiment[17,18,37]. With an ever-expanding repertoire of techniques to manipulate the genome, the mouse is a powerful tool to model the disruption of a specific human gene, and to analyse the behavioural and pathological outcome. Combining this resource with environmental intervention has only just begun, yet the first results are demonstrating that gene-environment interaction studies are possible and can generate valuable results.

## EXAMPLES OF GENE-ENVIRONMENT MODELS IN SCHIZOPHRENIA

### Snap-25 Mutant, *Blind-Drunk*

Random mutagenesis is one method that has been successfully harnessed in recent years to identify novel mouse models of behavioural disorders[38,39]. We initially characterised the *blind-drunk* (*Bdr*) dominant mutant from a phenotype-driven mutagenesis screen due to its subtle, slow, ataxic gait. We went on to show that *Bdr* mice harbour a point mutation in *Snap-25*, leading to neurotransmission defects and a reduced PPI response caused by inefficient dissociation of the core SNARE exocytosis complex[40]. An important feature of the neurodevelopmental hypothesis of schizophrenia is that synaptic connectivity is disrupted during key stages of brain development, ultimately leading to defects in neurotransmission and synaptic plasticity later in life[41]. Therefore, proteins like Snap-25, essential for such fundamental processes, are likely to be involved[42]. Furthermore, SNAP-25 has been implicated in schizophrenia via linkage and pathological studies[42,43,44]; thus, we reasoned that *Bdr* would be an ideal model for analysing gene-environment interactions combined with a well-studied synaptic abnormality. We chose to adapt a variable prenatal stress protocol previously used in rats as the environmental insult, followed by a battery of neurodevelopmental and behavioural analyses[22]. We also considered potentially confounding maternal and stress hormone factors, and confirmed that the stress regime generated robust corticosterone enhancement without overtly influencing care of the developing litters. Prenatal stress elicited aspects of anxiety in the open field and elevated plus-maze in all offspring at adulthood, regardless of genotype, consistent with further studies[45]. More importantly, the PPI deficits described previously were significantly enhanced in *Bdr* mice from stressed dams and this effect could be reversed using the antipsychotic clozapine, demonstrating predictive validity of the model. Social activity was also analysed using the three-compartment social testing apparatus[46]; time spent interacting with a stranger mouse confined to a small cage compared to an identical, but empty, cage (sociability) is analysed, followed by the time spent with a novel, confined, intruder mouse compared to the original stranger (social novelty). *Bdr* mice from nonstressed dams or wild-type mice from stressed dams showed no social deficits, but mutants from stressed mothers showed a profound lack of sociability in both parts of the test[45]. By contrast, wild-type littermates showed no significant PPI or social deficits in response to the chosen prenatal stress protocol. This work was the first to demonstrate that prenatal environmental stress combined with a synaptic genetic lesion could generate new and previously unseen schizophrenic endophenotypes.

### DISC1 Mutants

Since the discovery of a number of promising schizophrenia candidate genes and related pathways, a slew of targeted mouse mutants and knock-outs have been generated; their details and relative merits as disease models have been extensively reviewed elsewhere[47]. As such, they are obvious candidates for gene-environment studies. Disrupted in schizophrenia 1 (DISC1) is one such gene that was originally identified in a Scottish schizophrenia pedigree in 1997[48]. A number of mouse models have since been generated, including transgenic mice that constitutively express dominant-negative mutant human DISC1 (DN-DISC1)[49], and an inducible mutant that displays some sex-specific effects on learning and memory, and reduced dendritic arborisation in cultured cortical neurons (mhDISC1)[50]. Recent work using these models has focussed on the significance of immune activation in the psychopathology of schizophrenia. For example, to examine the effects of prenatal infection, neonatal polydI:dC injection has been studied in the inducible mhDISC1 mutant[51]. Mutant offspring from polydI:dC-injected dams showed an interaction between environmental and genetic factors, with alterations in anxiety and social phenotypes. Not all behavioural paradigms were altered in this model, however, as PPI and learning and memory performance were unaffected. Quantitative pathological analysis revealed a decrease in the density of spines on granule cells of the hippocampus in transgenics due to prenatal polydI:dC injection, although surprisingly, MRI studies revealed an attenuation of the enlarged lateral ventricles previously observed in the mhDISC1 line. This potential discrepancy between the pathological changes and the behavioural data observed remains unexplained. The use of an inducible DISC1 transgene meant that the timing of expression could also be assessed in relation to immune activation; if transgene expression was restricted to the embryonic/neonatal or postweaning stages, then no behavioural abnormalities were observed, demonstrating that continuous expression of mutant DISC1 was required for gene-environment interactions in this model.

## Complexin Mutants

Complexins (*Cplx*) are small presynaptic SNARE-interacting proteins that modulate neurotransmission, although presumably due to functional compensation, *Cplx2* knock-out mice only show a mild behavioural phenotype[52]. With some parallels to SNAP-25, in addition to association studies linking *CPLX* genes to the disease[53], *CPLX1* expression has been shown to be decreased in schizophrenic postmortem brains[54]. In an early attempt to combine postnatal psychological stress with *Cplx2* genetic lesions, maternal deprivation was carried out on knock-out mice followed by electrophysiological and some limited behavioural analysis[55]. This study showed that *Cplx2* null animals subjected to maternal deprivation stress showed a reduction in the induction of long-term potentiation (LTP), a measure of synaptic plasticity, as well as impairments in the Morris water maze learning and memory paradigm. Neurotrauma has also been described as a potential risk factor for schizophrenia[56]; in particular, damage to the parietal cortex[57]. A second more recent study applied a mild postnatal cortical lesion event to *Cplx2* knock-outs[58] to model this phenomenon. Mice subjected to the brain lesion did not show any significant behavioural abnormalities; however, the combination of the genetic and environmental factors caused both PPI and water maze memory deficits, in addition to an increased sensitivity to the NMDA antagonist MK-801 in tests of hyperactivity. MRI data also revealed a decrease in hippocampal volume in lesioned *Cplx2* knock-outs. These data again illustrate that combining genetic and nongenetic factors generate valuable correlates for neuropsychiatric disease.

## Reelin Mutants

Studies of *reelin*, a key neurodevelopmental gene, led to behavioural and pathological characterisation of the both homozygous (RL) and heterozygous (HZ) *reelin* knock-outs. The HZ line is of particular interest as it displays a range of endophenotypes and subtle neuroanatomical defects related to neuropsychiatric disease[59]. To examine the relationship between dopamine turnover and neurodevelopment, RL and HZ mice were prenatally exposed to chlorpyrifos-oxon (CPF-O), an acetylcholinesterase inhibitor. Interestingly, there appeared to be a somewhat complex dosage effect of the observed gene-environment interaction. For example, the normally reduced levels of ultrasonic vocalisation (USV) in RL week-old pups was reversed by CFP-O injection, whereas developmental retardation as measured by the righting reflex in RL mice was significantly impaired by prenatal CFP-O[60,61]. In a contrasting approach, maternal separation or handling during the first postnatal week was applied to *reelin* mutants. Somewhat surprisingly, once again, there was a positive synergistic effect of the perinatal stressor; only nonseparated RL pups showed markedly reduced levels of USV, while wild-type and HZ mice showed no difference in vocalisation between the maternal care conditions[62]. Why the lack of the *reelin* gene appeared to guard against the effects of adverse perinatal factors is unclear, although these data reinforce the observation that new and unexpected phenotypes can arise from gene-environment interactions.

## Mouse Strain Variability

It is also important to note that the innate differences between inbred mice strains have been harnessed to great effect to identify quantitative loci (QTLs) responsible for specific behaviours[63]. However, very few studies to date have combined this resource with environmental manipulation. As a model of immune challenge in early postnatal life, interleukin-1a (IL1-a) was administered to neonates from a range of mouse strains, with DBA/2 showing the most pronounced behavioural changes, including decreased PPI responses, yet increased locomotor activity[64]. With the full genome of multiple mouse strains near completion (see: http://www.sanger.ac.uk/resources/mouse/genomes/), understanding the subtleties of genetic variation in relation to such data will be an interesting task to complement studies on engineered mouse mutants.

## CONCLUSIONS AND FUTURE PROSPECTS

Modelling a human psychiatric disorder in rodents is obviously limited by the clinical symptoms that can be practically assessed experimentally. This raises the issue of justifying the validity of the model, which in itself can prove difficult in a disease where little is known about the aetiology and pathophysiology. Clearly, features of schizophrenia such as hallucinations and other thought disturbances cannot be measured in mice or rats. Therefore, in terms of face validity, it has been necessary to focus on neurobehavioural phenotypes and endophenotypes that address specific schizophrenia-related symptoms such as cognition and latent inhibition[65,66]. In addition, the processing of auditory information as assessed by PPI is a well-characterised paradigm that is not only conserved across mammalian species, but also provides some useful aspects of predictive validity[67,68]. However, as with all behavioural tests, there are caveats to the interpretation of results, including sex-specific differences combined with the potential confound of hearing disabilities[69]. Thus, with the limited scope and specificity of individual endophenotypes, few if any conventional mouse genetic models can supply both face and predictive validity in concert with neuroanatomical alterations that combine to generate a truly multifaceted model of psychiatric disease.

Consequently, it may be tempting to assume that the construct validity of a mutant rodent model of schizophrenia is instantly improved by adding an environmental factor into the mix. Whereas it should be recognised that such experiments are an important step forward in generating valuable new models of neuropsychiatric disease, as discussed above, there is currently a myriad of parameters to consider that individually can cause behavioural phenotypes that may mask or confound interpretation of the data. The subtleties of influencing rodent behaviour by altering their environment are also highlighted by work on environmental enrichment, which has been shown to essentially reverse the adverse effects of prenatal stress[70]. Indeed, such methods are now being extended to mouse mutant models of behavioural disorders[71]. Furthermore, the selection and validity of rodent pre- and postnatal stress models is still under debate, particularly when considering that many of the environmental risk factors translated from human studies are not specific to mental health susceptibility[72]. This also raises the issue that if the environment is so critical to neurodevelopment and ultimately adult behaviour, generating and comparing genuinely valid models of neuropsychiatric disease requires well-controlled and broad-based behavioural and pharmacological testing[73]. Standardising housing and screening apparatus is clearly not always practical, although some advocate these methods as a practical way of removing environmental bias in behavioural neuroscience[74].

It may seem a daunting task to model a highly complex disorder such as schizophrenia with few consistent genetic or neuropathological features. Yet, the relatively new field of research into gene-environment interactions in psychiatric disease is clearly gathering pace. As new genetic manipulation tools are improving the validity of the mouse models that are available, behavioural, pathological, and electrophysiological methodologies are constantly being refined. Added to this is the undoubted exponential growth in sequencing data that is likely to reveal many new genetic variants that will play predominantly minor roles in susceptibility to psychiatric disease[75]. In parallel, attempts are being made to carry out prospective studies on pregnant women and their offspring in order to gather a more comprehensive dataset on environmental factors and neuropathological outcomes. A recent example of this is the ongoing Project Ice Storm, where severe cold weather in southern Quebec, Canada affected over 3 million people in 1998 over a wide geographical area and social range[76]. In addition to following childhood development, MRI data are being collated[10], providing valuable information associating a specific prenatal event to alterations in brain development and structure.

It is accepted that a perfect rodent correlate of psychiatric disease will never exist[65,77]. However, with experiments to date demonstrating that previously unseen and enhanced behavioural phenotypes can be observed in mutant mice subjected to environmental stress, such models will become an increasingly important tool to identify the mechanisms that underlie the synergy of multiple genetic and nongenetic factors in mental health.
